# Influence of Content in *D* Isomer and Incorporation of SBA-15 Silica on the Crystallization Ability and Mechanical Properties in PLLA Based Materials

**DOI:** 10.3390/polym14061237

**Published:** 2022-03-18

**Authors:** Tamara M. Díez-Rodríguez, Enrique Blázquez-Blázquez, Ernesto Pérez, María L. Cerrada

**Affiliations:** Instituto de Ciencia y Tecnología de Polímeros (ICTP-CSIC), Juan de la Cierva 3, 28006 Madrid, Spain; t.diez@ictp.csic.es (T.M.D.-R.); enrique.blazquez@csic.es (E.B.-B.); ernestop@ictp.csic.es (E.P.)

**Keywords:** PLLA, *D* isomer, SBA-15 composites, α’ and α forms, cold crystallization, microhardness, long spacing

## Abstract

Two *L*-rich polylactides (PLLA) with distinct contents in *D* isomer and their composites with an intermediate amount of mesoporous Santa Barbara Amorphous-15 (SBA-15) (about 9 wt.%) particles were attained by melt extrusion for the evaluation of the effect of content in *D* isomer and incorporation of mesoporous silica on the structural PLLA features and on their ultimate mechanical performance. For that, samples have been crystallized under dynamic and isothermal tests (from the melt and from the glassy states). The results from DSC and X-ray diffraction show obtainment of the pure α’ and α modifications at different intervals of crystallization temperature depending on the *D* steroisomer amount of the PLLA used. Furthermore, several phase transitions are observed depending on the crystallinity reached and the polymorphs developed during the isothermal crystallization from the glass: an additional cold crystallization, the α’/α transformation and the subsequent melting process, appearing all of them at temperatures clearly dependent on the *D* content. Rigidity, measured through microhardness in amorphous samples, is also affected by the *D* isomer and the presence of SBA-15 particles. Reinforcement effect of mesoporous silica is relatively more important in the matrix with the highest *D* content.

## 1. Introduction

Poly(*L*-lactide) (PLLA) has received a great interest in the past two decades as a renewable and biodegradable polymeric material because of the concerns about sustainable development and environmental pollution issues connected with the massive uses of conventional petroleum-based polymers. Its potential to become a commodity is based on its high modulus, easy processability, excellent biocompatibility and recyclability, compared with other biodegradable materials. The extensive applications of PLLA have been, however, considerably prevented by its intrinsic slow crystallization kinetics [1,2], poor heat resistance and brittleness [3,4]. All of these shortcomings have been attempted to be overcome by several approaches [5,6,7,8,9,10,11]. Thermal treatment above glass transition temperature (*T_g_*) (also designated as recrystallization) is a useful route, applied during or after its manufacturing, because it leads to a crystallinity increase, an enlargement in Young’s modulus and in heat distortion temperature as well as an improvement in impact resistance and tensile strength without altering its molecular mass [12,13,14,15].

Moreover, several investigations have been focused on the improvement of PLLA overall crystallization kinetics [16] by the inclusion of plasticizers to boost chain mobility or by the incorporation of nucleating agents to increase nucleation density. CaCO_3_, TiO_2_, talc, calcium lactate, carbon nanotubes, graphene oxide, nanoclay, and hydrazides have been commonly used as nucleants [16,17,18,19,20,21,22,23,24,25]. Ordered mesoporous silicas can be an appropriate option to induce nucleation since they can interact with PLLA chains from interior of the empty pores existing in their arrangements or exterior of the particles. They are thermal and chemically stable materials with uniform pore size, pore distribution, high surface area and high adsorption capacity. Size and shape of these silica particles together with structure of pores can be tuned by controlling synthetic parameters like reaction time, temperature, surfactant concentration, amount of silicon source, or changing calcinations conditions [26,27]. These silicas are useful in several applications, such as environmental, biomedical, energy, and as catalyst supports. The Mobil Composition of Matter No. 41 (MCM-41) and Santa Barbara Amorphous-15 (SBA-15) are their best-known members. They comprise hollow channels ordered in hexagonal frames [26,27]. Pore size is the main difference between them, diameter ranging from 2 to 4 nm in the former [26] while varying from 5 to 10 nm in the latest [27].

Similar to other polyesters, PLLA is capable to develop several polymorphs depending on the crystallization protocol applied [16]. Crystallization from the melt of PLLA at temperatures higher than about 120 °C or from solution under regular conditions turns out in formation of its most stable and common polymorph, the α form, in which segments with a 10_3_ helical chain conformation are packed into an orthorhombic unit cell [28,29,30]. Development of α’ crystals is observed if melt crystallization takes place at temperatures lower than about 120 °C, these crystallites exhibiting a markedly specific enthalpy of melting lower than the α-phase [31]. Transformation from the α’ to the α polymorph has been described in the DSC curve during the heating process through a small exotherm prior the dominant melting process [17,32,33,34]. Nevertheless, the α’-crystals can be completely melted without previous phase transition into the α-polymorph by heating faster than 30 K/s [35]. These α’ entities were initially considered as conformationally disordered α-crystals [33,34,36,37] although Wasanasuk and Tashiro [38] proposed that this α’ lattice was a crystal modification independent of the α-form, and not simply the α form with some degree of structural disorder. Another different polymorph, the β form, can be attained at high draw ratio and high drawing temperature and is known to take a left-handed 3_1_ helical conformation [30,39,40], whereas the γ form, as described by Cartier et al. [41], is achieved by epitaxial crystallization. Furthermore, the formation of meso-crystal has been also reported [42,43,44]. Nevertheless, foremost interest has been focused on the α and α’ crystals from both academic and industrial standpoints since they are the only ones growing upon usual processing conditions.

Another important characteristic that exerts a great effect on PLLA properties is the presence of *D*-lactic acid units in its macrochains since they provoke a break in its chain regularity and reduce the subsequent melt-crystallization rate. The monomer, lactic acid, contains an asymmetric carbon atom and when it is polymerized leads to a mixture of enantiomers (*L*-lactic acid or *D*-lactic acid). Two routes are employed for its synthesis: polymerization by condensation of lactic acid monomer or by ring-opening of lactide [45], which is its cyclic dimer. This latest is the procedure used in the production for commodity applications, having allowed cost-efficient PLLA manufacture of high-molar mass. Commercial PLLA grades with the most industrial relevance, commonly designated as poly(lactic acid) (PLA), usually consist of an *L*-rich mixture where *D*-lactide content, which comes from either the *DD*-lactide or the *LD* meso-lactide, ranges from 1 to 10% molar composition. These polymeric resins are semicrystalline if contain more than 93% of *L*-lactic acid while those with an amount of 50–93% in *L*-lactic acid are completely amorphous [4]. Therefore, the presence of both *L* and *D* isomers in the PLLA macrochains makes it a random copolymer, the amount in the minority *D* isomer being a key variable for their crystalline details. It has been reported that spherulite growth rate and crystallization rate decrease with rising *D*-isomer content in the PLLA chain [46,47]. Furthermore, the critical cooling rate to suppress crystallization is lowered from 0.5 K/s in the case of the PLLA homopolymer to 0.05 K/s for the random copolymer with 4% *D*-isomer content [47]. Moreover, observation of a reduction of the rate of formation of homogenous crystal nuclei due to the presence of *D*-isomer in the chain allows concluding that these defects may already be excluded from the ordered structures at this early stage of the crystallization process. Nevertheless, there are not many investigations dealing with changes in crystalline polymorphs and the derived properties in PLLA with different content in *D*-isomers since most of the studies up to now only detail the PLLA crystallization aspects [46,47].

The goal of this research is to evaluate the effect of incorporation of a similar amount of mesoporous SBA-15 into two PLLA matrices with different *D*-lactic acid composition, focusing attention on their melting and crystallization behavior, the polymorphs developed, together with their influence in the ultimate mechanical response. The size of pores in SBA-15, in addition to the fact that it is commercial silica, is the reason for its selection instead of other mesoporous silica materials. SBA-15 particles could promote insertion of the polymeric macrochains within the hollow interior of silica nanostructures and, thus, favor interactions between both components. The novelty of this research consists of comparing the effect that the addition of these mesoporous particles exerts on PLLAs with different content in the *D*-isomer. Moreover, they could be readily decorated, fact that could allow the incorporation of different interesting functionalities to the polymeric matrix in a further stage, leading to multipurpose PLLA materials. The present composites have been obtained by melt extrusion, an environmentally friendly, and cost-effective transformation approach because it does not require using solvents. Several techniques have been employed in this investigation, including nuclear magnetic resonance (NMR), size exclusion chromatography (SEC), transmission electron microscopy (TEM), scanning electron microscopy (SEM), thermogravimetry (TGA), differential scanning calorimetry (DSC), X-ray experiments with conventional and synchrotron radiation, together with microhardness tests.

## 2. Materials and Methods

### 2.1. Materials

Two commercial *L*-rich polylactides (PLLA) from NatureWorks^®^ (Minnetonka, MN, USA) are used in this investigation. Their grades are named PLA Polymer 3051D and Ingeo™ Biopolymer 6202D, with a density of 1.25 and 1.24 g/cm^3^, respectively.

The mesoporous SBA-15 silica was purchased from Sigma-Aldrich (St. Louis, MO, USA) (specific surface area, *S*_BET_ = 517 m^2^/g; total pore volume, *V_t_* = 0.83 cm^3^/g; average mesopore diameter, *D_p_* = 6.25 nm [48]) and was used as received.

### 2.2. Composites Preparation by Extrusion and Film Processing

Composites with a relatively high content in SBA-15 particles were prepared by melt extrusion at a rate of 60 rpm in a corotating twin-screw microextruder (Rondol, Rondol Industrie, Nancy, France). Both the silica particles and PLLA polymers were dried previous to extrusion. The former were dried under vacuum at 100 °C for 24 h. The PLLA homopolymers were initially dried in an oven at 100 °C for 20 min followed by drying under vacuum at 85 °C for 2 h. A screw temperature profile of 125, 160, 190, 190, and 185 °C was employed in the extruder from the hopper to the die. The length-to-diameter ratio was 20:1.

Films were processed by compression molding in a hot-plate Collin press (Collin GmbH, Maitenbeth, Germany). Initially, the material was maintained at a temperature of 185 °C and at a pressure of 30 bar for 6 min. Afterward, a cooling stage was applied at a relatively fast rate of around 80 °C/min and at a pressure of 30 bar for 4 min from the melt to room temperature to the different materials. These original compression-molded films were totally amorphous.

### 2.3. Characterization of Pristine PLLAs and Their Composites with SBA-15

The weight-average molecular weight (*M_w_*), number-average molecular weight (*M_n_*), and polydispersity index (*M_w_/M_n_*) of the two used PLLAs were measured by Size Exclusion Chromatography (SEC) with a chromatographic system (Waters Division Millipore) (Milford, MA, USA) equipped with a refractive index detector. Tetrahydrofurane (99.9%, Aldrich (St. Louis, MO, USA)) was used as the eluent at a flow rate of 1 mL/min at 35 °C. Styragel packed Waters columns (HR 1, 5 µm, 7.8 mm × 300 mm) were used. Molecular masses of polymers were referenced to PS standards.

The optical purity was measured by ^13^C NMR for the two commercial PLLA grades. They were dissolved in deuterated chloroform (Aldrich, St. Louis, MO, USA) (10% weight/volume). To calibrate the scale of chemical displacement to 0 ppm, two drops of tetramethylsilane (TMS) (Aldrich, St. Louis, MO, USA) were added. ^13^C NMR spectra, decoupled ^1^H, at room temperature were recorded with a 10 mm probe on a Varian Inova 400 spectrometer (Palo Alto, CA, USA). ^13^C NMR spectra were achieved by Fourier transform after Gaussian multiplication.

Materials studied have been labeled as PLLA2 and PLLA4 for Ingeo6202D and PLA3051D, respectively, according to the ^13^C NMR results. Moreover, their composites are designated as PLLA2SBA8 and PLLA4SBA9, indicating the last number in their names the corresponding weight content in SBA-15 particles determined by TGA analysis (see below).

Morphological information of the mesoporous particles were attained by transmission electron microscopy (TEM). Measurements were performed at room temperature in a 200 kV JEM-2100 JEOL microscope (JEOL Ltd., Tokyo, Japan). The SBA-15 silica was dispersed in acetone in an ultrasonic bath for 5 min and then deposited in a holder prior its observation.

Dispersion of SBA-15 within the PLLA matrix was evaluated by high resolution field emission scanning electron microscopy (FESEM). Experiments at room temperature were carried out in a S-8000 Hitachi equipment (Hitachi Co., Tokyo, Japan) in different cryo-fractured pieces of composites. Those thin portions, of around 40 nm, were cut at −120 °C by cryo-ultramicrotomy (Leica EM UC6, Leica Microsystems GmbH, Wetzlar, Germany) and deposited in a holder.

Thermogravimetric analysis (TGA) was made in a Q500 equipment of TA Instruments (New Castle, DE, USA) at a heating rate of 10 °C/min under nitrogen or air atmosphere. The actual SBA-15 content incorporated into the composites prepared by extrusion is determined by this technique, which is calculated as an average of values.

Calorimetric analyses were performed in a TA Instruments Q100 calorimeter (New Castle, DE, USA) connected to a cooling system and calibrated with different standards. The sample masses were around 3 mg. A temperature interval from −30 to 180 °C was studied at a heating rate of 10 °C/min. A value of 93.1 J/g was used as fusion enthalpy of perfectly α crystalline material [17,49] for determination of crystallinity.

Real-time variable-temperature small-angle (SAXS) and wide-angle (WAXS) experiments were performed using synchrotron radiation in beamline BL11-NCD-SWEET at ALBA (Cerdanyola del Valles, Barcelona, Spain) at a fixed wavelength of 0.1 nm. A Pilatus 1 M detector (Dectris Ltd., Baden-Daettwil, Switzerland) was used for SAXS (at a distance of around 300 cm from the sample) and a Rayonix one (Rayonix, Evanston, IL, USA) for WAXS (at about 14.6 cm from the sample, and a tilt angle of around 29 degrees). A Linkam Unit (Waterfield, UK), coupled to a cooling system of liquid nitrogen, was utilized for the temperature control. The calibration of spacings was attained by means of silver behenate and Cr_2_O_3_ standards. The initial 2D X-ray images were transformed into 1D diffractograms, as a function of the inverse scattering vector, *s* = 1/*d* = 2 sin *θ*/*λ*, by means of pyFAI python code (ESRF), modified by ALBA beamline staff. Film samples of around 5 × 5 × 0.1 mm^3^ were used in the synchrotron analysis.

A Vickers indentor attached to a Leitz microhardness tester (Leitz GmbH, Oberkochen, Germany) was employed for microindentation measurements at 23 °C. A contact load of 0.98 N and a time of 25 s were employed. Microhardness, *MH*, value (in MPa) was calculated according to the relationship [50,51]:

$$MH=2\sin68^{\circ }\left(\frac{P}{d^{2}}\right)$$

where *P* (in N) is the contact load and *d* (in mm) is the diagonal length of the projected indentation area. Diagonals were measured in the reflected light mode within 30 s of load removal, using a digital eyepiece equipped with a Leitz computer-counter-printer (RZA-DO).

## 3. Results

Table 1 lists the values obtained from ^13^C NMR spectra in the determination of mol percentage in *D*-lactic isomer for the two PLLA matrices used in this research. The methine proton and the methine carbon are best suitable for stereosequence analyses, as suggested in the literature [52], and ^13^C NMR has been the choice selected considering its better resolution. There is an important difference between the molar content in *D*-isomer for the two PLLAs, fact that presumably will affect considerably their crystallization capability and, probably, their whole crystalline characteristics exhibited. In spite of the similar SBA-15 content incorporated to both PLLAs, their effect could be rather different depending on the polymeric matrix considered.

Furthermore, molecular weight has been estimated on both PLLAs after extrusion was applied and the final pristine polymeric matrix has been obtained. It is seen the great similarity found in the *M_w_* presented by the two PLLAs. Accordingly, the content in *D*-isomer is the only microstructural parameter with a great influence in the final crystalline structure and, thus, in the ultimate performance.

**Figure 1:**
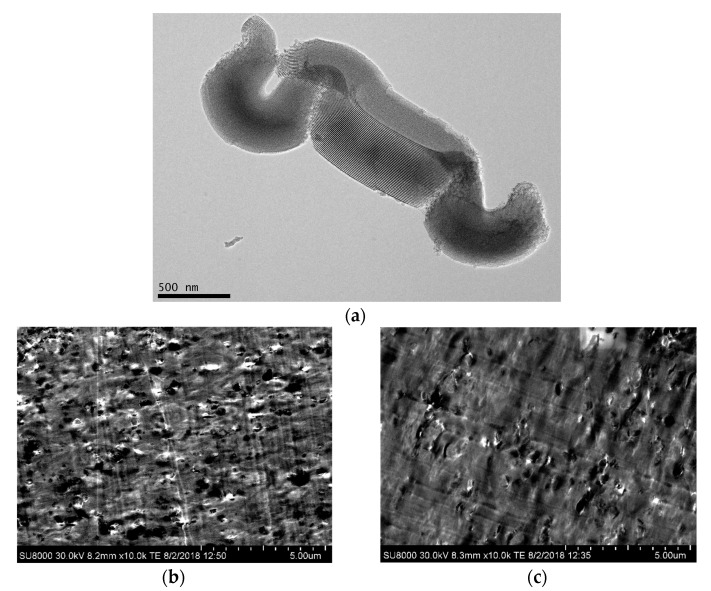
(**a**) TEM micrograph for SBA-15 silica at a scale bar of 500 nm. FESEM images in the two composites: PLLA2SBA8 (**b**) and PLLA4SBA9 (**c**), at a scale bar of 5 μm.

Figure 1a shows a TEM micrograph for the SBA-15 particles used. Their characteristic vermicular elongated shape is displayed in the picture with an average size of 0.9 μm long and 350 nm wide. A well-defined, ordered, and uniform channel arrangement is clearly noticeable, which constitutes the interior part of the particles and leads to morphological details consisting in a hexagonal structure in the transverse direction.

Pictures depicted in Figure 1b,c correspond to FESEM micrographs for the composites PLLA2SBA8 and PLLA4SBA9. It is noted a good distribution of mesoporous SBA-15 in the two PLLA matrices and the absence across the films of bulky silica domains.

Some agglomerates are, however, seen but their sizes are not extremely large and the proportion in which they are found is rather negligible. A suitable contact appears to take place at the interfaces between mesoporous particles and the two PLLA polymeric matrices.

The SBA-15 silica is extremely hydrophilic and PLLA undergoes easily hydrolytic degradation. This PLLA decomposition is noteworthy mainly at high temperatures, leading to a lessening of molecular mass and, subsequently, to the loss of properties. It is, then, imperative to analyze the effect of SBA-15 particles on the thermal stability of PLLA because these materials are obtained at high temperatures by extrusion. Furthermore, mesoporous silicas are characterized to be, sometimes, used as catalysts during polymer decomposition. This role was reported in the polyethylene degradation under an inert atmosphere with and without the presence of MCM-41 particles [53]. The efficacy of mesoporous MCM-41, when used as a promoter of the decomposition of polyolefins to liquid fuels had also been described [54]. This character of boosting degradation was also observed for in situ polymerized polyethylene-based composites when MCM-41 particles, either neat or decorated with undecenoic acid or silanes, were employed as catalyst support and as fillers [55,56,57].

**Figure 2:**
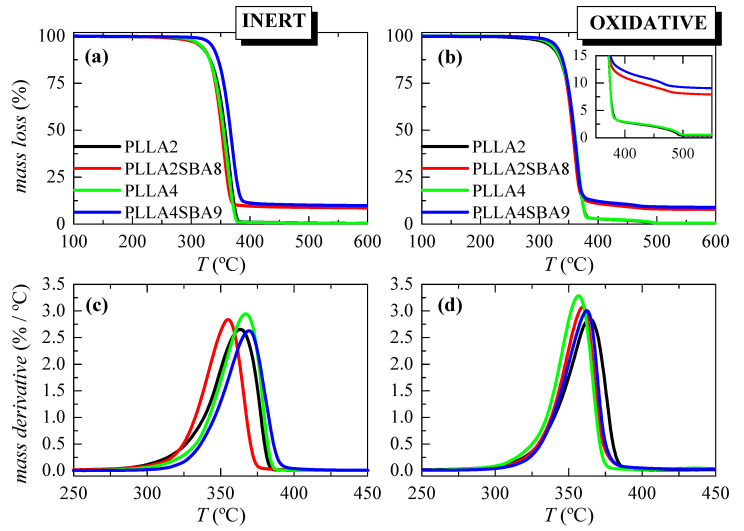
TGA curves under nitrogen (left) and air (right) atmospheres for the pristine PLLA matrices and their SBA-15 composites attained by extrusion ((**a**,**b**) representations); and their DTGA curves ((**c**,**d**) representations).

Figure 2 displays the TGA curves and their derivatives (DTGA) under nitrogen and air environments for these extruded composites. It is noticed that inert decomposition occurs through a unique stage ranging from 275 to 400 °C in these two PLLAs. The addition of mesoporous SBA-15 does not change much how the process takes place or the temperature interval. A displacement to a higher temperature is, however, noted for the 10% mass loss in PLLA4SBA9 as detailed in Table 2. It is noticeable that the lower temperature at the maximum is exhibited by the PLLA2SBA8 composite while the higher one is observed in PLLA4SBA9. Moreover, the degradation process from the DTGA curves becomes narrower in the two composites.

Two degradation processes are exhibited in the TGA curves under air. The main decomposition step occurs at the lower temperature and implies most of the mass loss, around 97%. Moreover, the secondary process takes place just after the major mechanism ends. The presence of SBA-15 does not exert a considerable influence in the main PLLA degradation stage, independently of the *D*-lactide content in the PLLA matrix. Nevertheless, mesoporous particles within PLLA2SBA8 lead to a small shift to lower temperatures in the maximum degradation compared with PLLA2 while PLLA4SBA9 shows a slightly higher *T*_max_ regarding its PLLA4 matrix. Furthermore, the temperature range at which the minor process occurs is narrowed, as seen from the inset.

These results demonstrate that the incorporation of SBA-15 silica in these PLLA matrices does not play a clear role as a degradation catalyst in PLLA2SBA8 and PLLA4SBA9 composites. Moreover, the precise content of the SBA-15 added is determined from these curves obtained under the two atmospheres. Results are also detailed in Table 2 and an average has been estimated. The values determined are quite similar indicating indirectly the homogeneity in the dispersion of the SBA-15 silica within the two PLLA matrices.

**Figure 3:**
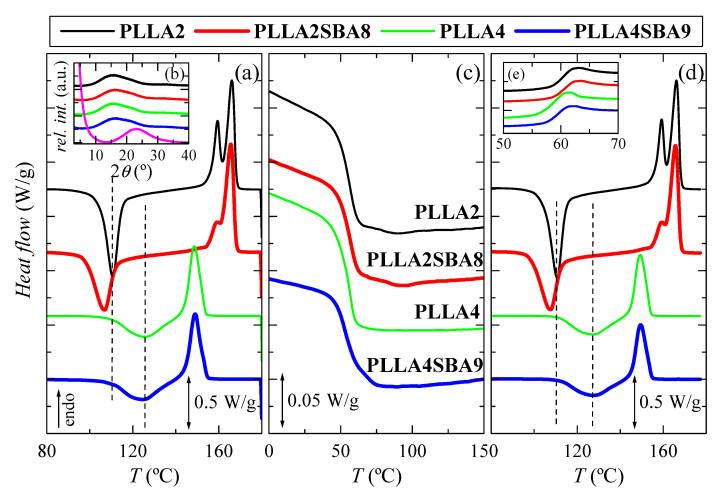
DSC curves carried out at 10 °C/min for the two PLLA analyzed, PLLA2 and PLLA4, and their hybrids with SBA-15, PLLA2SBA8 and PLLA4SBA9: (**a**) first heating; inset (**b**) X-ray patterns at room temperature in the wide-angle interval; magenta profile is the one for the pristine SBA-15 particles; (**c**) cooling from the melt; (**d**) second heating, and inset (**e**) glass transition interval during second heating.

Figure 3 depicts the phase transitions found in the just processed films for the neat PLLAs and their hybrid materials with SBA-15, PLLA2SBA8 and PLLA4SBA9, during the first heating, cooling and second heating at 10 °C/min. This information is required in polymers to understand their thermal properties, but it is even more significant in PLLA since it undergoes physical aging below its glass transition temperature (*T_g_*) and its crystallization occurs slowly, involving both characteristics an important effect in its further dimensional stability. Analysis will be mainly centered on crystallization because presence of SBA-15 silica could act as a nucleating agent and could affect the ordering capacity of PLLA macrochains.

Figure 3a shows the first heating run. Glass transition (not displayed) takes place at approximately 60 °C in all the specimens, i.e., the addition of mesoporous silica does not seem to affect its location much, as also can be deduced from the cooling represented in Figure 3c. At higher temperatures, a noticeable cold crystallization process is observed in Figure 3a for all of the PLLA based materials, either pristine or composite. An important difference is found in the position of cold crystallization temperature, *T_cc_*, for both neat PLLAs. It appears at 110.5 °C in PLLA2 while its location is 126.5 °C in PLLA4. Furthermore, this process is much broader in the latest than in the PLLA2, indicating the much more hindrance encountered for crystallizing in the PLLA4. This feature can be only ascribed to the different content in *D*-enantiomer existing between PLLA2 and PLLA4, since this is the only distinctive microstructural parameter in these two matrices because of their molecular masses and polydispersity indices are rather analogous (see data in Table 1). That difference becomes even larger when SBA-15 particles are incorporated since *T_cc_* is found at 106.5 °C in the PLLA2SBA8 composite and in PLLA4SBA9 at 124.5 °C. It is also remarkable that crystallization width is increased in PLLA2SBA8 compared with that noted in the neat PLLA2. On one hand, therefore, SBA-15 particles play the role of nucleating agent allowing crystallization to occur at lower temperatures on heating and, on the other hand, their presence makes crystallization more heterogeneous. The effect of SBA-15 as nucleant is also observed when PLLA4SBA9 is compared with PLLA4. It is expected in both PLLAs and in their composites that the α form is the main polymorph developed during this cold crystallization since it occurs at temperatures above 100 °C. This aspect will be discussed below.

After the exothermic process ends, a melting is initiated. In PLLA2 and PLLA2SBA8 specimens, a bimodal endotherm is noted, whose main melting temperature, *T_m_*, appears at about 166 °C, while a unique peak seems to be observed in PLLA4 and PLLA4SBA9, whose maximum is located at 149 °C (this melting peak, however, may have two components, but with very close temperatures). Again, a great difference between both pristine PLLAs is noted in terms of the location of their *T_m_* as well as in that of their respective composites, characteristics similar to those mentioned for *T_cc_*. Content in *D* isomer of 4.2 for PLLA4 hampers significantly the formation of crystallites compared with that ordering in the PLLA2 matrix with a molar composition in *D* enantiomer of 2% and, consequently, an important delay of crystallization is observed in PLLA4 and its composite. This implies that those crystals are less perfect and much thinner than those in PLLA2 and PLLA2SBA8, and, accordingly, they melt at significantly lower temperatures.

Values of enthalpies implicated in the cold crystallization and the subsequent melting process are equivalent, leading to a zero neat total enthalpy of melting. This fact indicates the complete amorphous nature of PLLA, after the quench applied during film processing, in the pristine polymers, and when they act as matrices in the PLLA2SBA8 and PLLA4SBA9 composites. This result agrees with those achieved from the X-ray diffraction measurements at room temperature, whose profiles, totally amorphous, are represented in the inset Figure 3b. The pristine SBA-15 is also completely amorphous at short range, as deduced from the magenta curve, exhibiting a wide reflection centered at around 2*θ* = 23°. Therefore, the PLLA2SBA8 and PLLA4SBA9 composites show an evident shoulder superimposed to the PLLA amorphous halo, which corresponds to the contribution of SBA-15 to the overall diffractogram. In addition to this amorphous halo, mesoporous SBA-15 displays its well-defined and ordered hexagonal arrangement [27] at low angles (not shown in Figure 3b), characterized by three main reflections indexed as (100), (110), and (200). These diffractions also appear in PLLA2SBA8 and PLLA4SBA9, although with much less intensity, indicating that the hexagonal structure of SBA-15 has been preserved in the composites during its incorporation by extrusion.

Figure 3c represents the process of cooling at 10 °C/min (Y scale has been magnified by a factor of 10 in relation to the melting curves). The unique transition clearly noted is the glass transition, which occurs at around 55 °C. Its position is not significantly altered by adding the mesoporous particles. In addition to *T_g_*, a rather small crystallization process is noticed in PLLA2 and its composite PLLA2SBA8. This crystallization is even less intense in PLLA4SBA9 taking place now in the vicinity of the beginning of its glass transition. SBA-15 role as nucleating agent deduced from the first heating run is again slightly evident.

Figure 3d shows the curves corresponding to the second heating process, after cooling at 10 °C/min, i.e., at a significantly slower rate than that used during initial film obtainment. Glass transition region is now represented in Figure 3e. It can be deduced that PLLA2 shows its *T_g_* at slightly higher temperatures than that for the PLLA4. Furthermore, the extent of physical aging in these samples is noticeably reduced in comparison with that noticed along with first heating. These specimens have been just cooled and there is no time for enthalpy relaxation to take place. Furthermore, it should be commented that no important changes are observed in the PLLA cold crystallization after the cooling at 10 °C/min compared with the initial specimens processed by compression since both are initiated from a practically complete amorphous state. After this cold crystallization, melting starts and its characteristics are the same as those discussed along the first heating process: a double peak in PLLA2 and PLLA2SBA8 specimens and a unique endotherm in PLLA4 and PLLA4SBA9 samples.

The important differences found between PLLA2 and PLLA4 together with the nucleating influence of SBA-15 silica in the PLLA2SBA8 and PLLA4SBA9 composites make a more exhaustive evaluation of the crystallization process necessary. Therefore, isothermal experiments have been carried out from the molten and the glass states.

**Figure 4:**
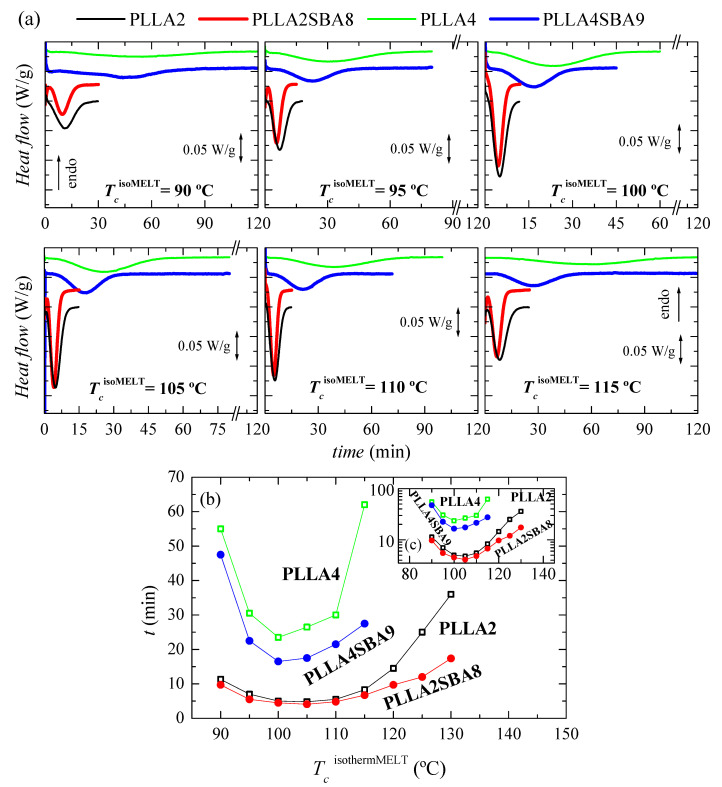
(**a**) Variation with time of heat flow for isothermal crystallization from the melt, *T_c_*^isothermMELT^, at different temperatures for PLLA2, PLLA2SBA8, PLLA4, and PLLA4SBA9. (**b**) Dependence of time for peak crystallization with the *T_c_*^isothermMELT^ in the PLLAs and their composites. (**c**) Time is represented in the logarithmic scale as a function of *T_c_*^isothermMELT^.

Figure 4a displays the processes of isothermal crystallization from the melt for the two neat PLLAs and their composites PLLA2SBA8 and PLLA4SBA9 at the distinct temperatures analyzed. It is particularly noted that incorporation of SBA-15 particles affects the PLLA crystallization, this being sped up independently of the content in *D* isomer existing at each PLLA matrix and of the *T_c_*^isothermMELT^ analyzed. Consequently, the crystallization is displaced to shorter times and becomes narrower in the composites at a given *T_c_*^isothermMELT^ in relation to their respective matrix.

A great effect of *D* isomer molar composition is also observed in the isothermal crystallization similar to what was seen in dynamic experiments. Crystallization is importantly hindered in PLLA4 and PLLA4SBA9 compared with that ordering process in PLLA2 and PLLA2SBA8. Rupture of chain regularity by the *D* isomer restricts the capability of crystal development. This lower ordering ability is not compensated by the incorporation of SBA-15 particles in spite of their nucleant role. It even seems that mesoporous silica nucleates more favorably in PLLA4 than in PLLA2, judging from the separation between the two curves (neat PLLA and composite) for the highest crystallization temperatures tested (130 °C for PLLA2 and 115 °C for PLLA4). Moreover, the fastest crystallization temperature for polymeric chains in the PLLA4 and PLLA4SBA9 is 100 °C while it is found at a slightly higher temperature, 105 °C, in the pristine PLLA2 and in PLLA2SBA8, as seen in Figure 4b,c.

**Figure 5:**
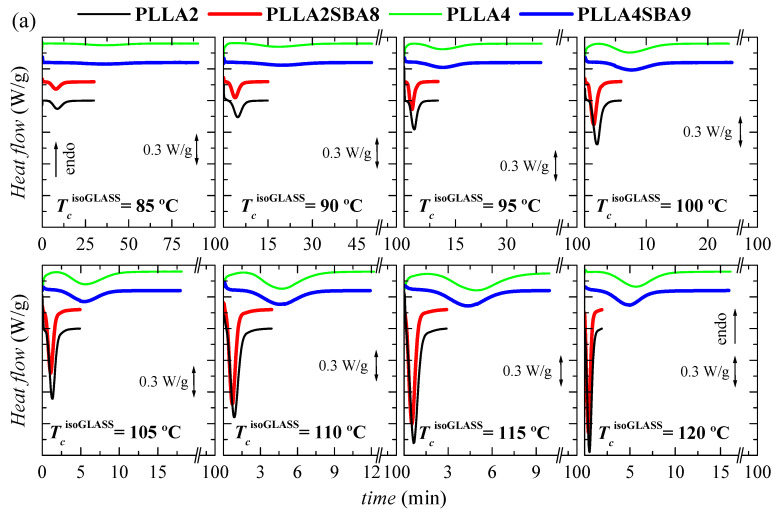

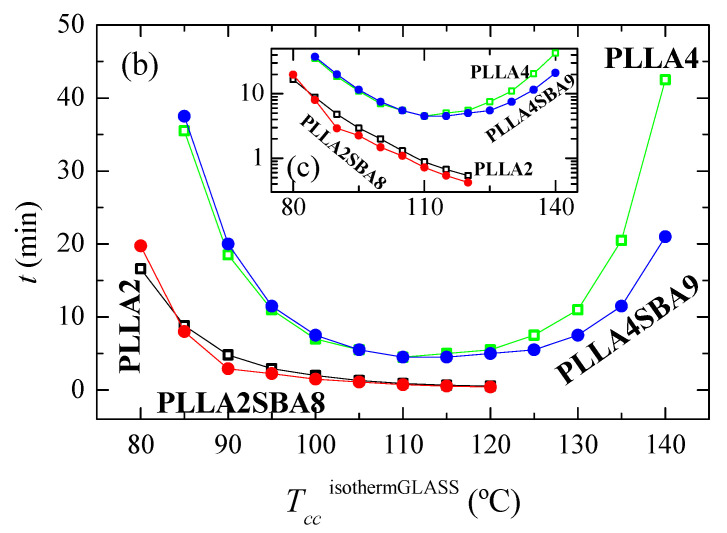
(**a**) Variation with time of heat flow for isothermal cold crystallization from the glass at the different temperatures, *T_cc_*^isothermGLASS^, for the several samples. (**b**) Dependence of time at which peak crystallization appears with the *T_cc_*^isothermGLASS^ in the two matrices, PLLA2 and PLLA4, as well as the composites PLLA2SBA8 and PLLASBA9. (**c**) Time as a function of *T_cc_*^isothermGLASS^ in logarithmic scale.

Further crystallization under isothermal conditions from the amorphous glass have been also carried out, taking into account the rather long times required for the crystallization from the melt. Figure 5 represents the results attained in a wide range of cold crystallization temperatures, labeled as *T_cc_*^isothermGLASS^, from 80 to 140 °C. 

As noted in Figure 5a,b, the formation of three-dimensional PLLA arrangements is again in PLLA2 and PLLA2SBA8 much faster than in PLLA4 and PLLA4SBA9. The nucleant influence of mesoporous silica is also observed at a given PLLA. This is noted at the whole temperature interval analyzed, with the exception of 80 °C, in PLLA2SBA8 although the difference is not very remarkable. The nucleating effect is more outstanding in PLLA4SBA9 at temperatures higher than 115 °C, where a minimum is clearly observed, which is related to the opposite characteristics shown in the crystallization rate by the nucleation and transport terms. The distinct trend seen in these two composites is associated with the fact that crystallization is hampered in PLLA4SBA9 regarding PLLA2SBA8 by the existence of a higher amount of *D* steroisomer in the PLLA4 matrix. Hindrance for the development of crystallites from the glass state is so great at temperatures below 115 °C in pristine PLLA4 that the influence of incorporation of SBA-15 is minimized.

Comparison of Figure 4b,c with Figure 5b,c allows deducing that, as expected, isothermal PLLA crystallization is much faster from the glass than from the molten state in all of the materials studied. In fact, the isothermal crystallization from the glass in the case of PLLA2 and PLLA2SBA8 becomes so fast that it cannot be measured above 120 °C. Moreover, the nucleating ability of SBA-15 particles becomes more significant when crystallization is started from the melt.

**Figure 6:**
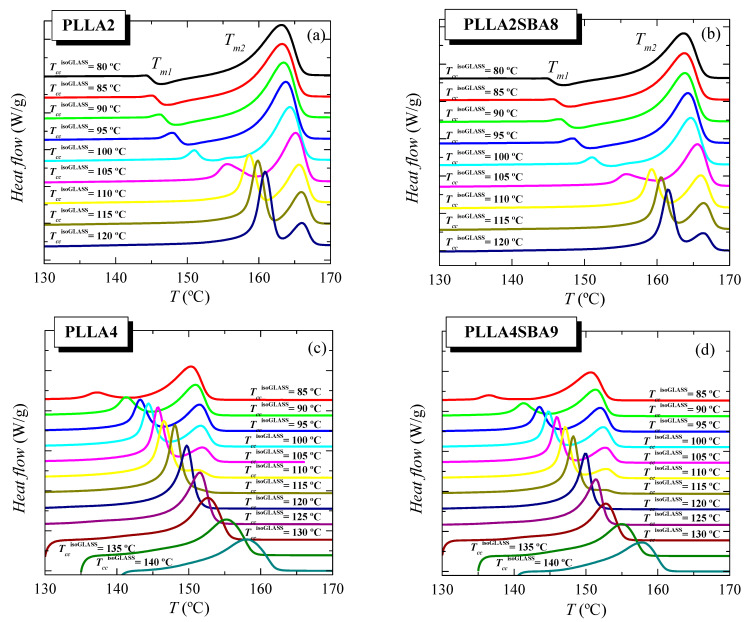
Melting behavior as a function of temperature used for isothermal crystallization from the glass, *T_cc_*^isothermGLASS^, for (**a**) neat PLLA2, (**b**) its composite PLLA2SBA8, (**c**) pristine PLLA4, and (**d**) its composite PLLA4SBA9.

Figure 6 shows the features observed during the further melting process after isothermal cold crystallization from the glass for the two PLLAs and their respective composites, PLLA2SBA8 and PLLA4SBA9. Firstly, focusing the attention on PLLA2 and PLLA2SBA8, two endothermic processes, whose maxima are labeled as *T_m_*_1_ and *T_m_*_2_, are observed in this *T_cc_*^isothermGLASS^ range. That located at the lowest temperature exhibits a little intensity and appears overlapped with a small exothermic process up to around 100 °C. This is ascribed to the α’/α-crystal transformation, as described previously in the literature [17,32,33,34]. Crystals developed during the isothermal cold crystallization in the interval of low temperatures are α’-type and these crystallites undergo on heating an α’ crystal → α crystal transition, leading to more perfect ordered entities. At *T_cc_*^isothermGLASS^ higher than 110 °C, this endothermic event becomes the most intense melting peak and undertakes a clear narrowing. Now, that formation of α’ lattice is not the most favorable but the α form. This melting peak, characterized by *T_m_*_1_, is now ascribed to the melting of the original α-type crystallites formed during the isothermal cold crystallization.

On the other hand, the melting process placed at the highest temperatures with a maximum at *T_m_*_2_ is very broad at the lowest *T_cc_*^isothermGLASS^ and its width decreases as cold crystallization takes place at superior temperatures. The enthalpy involved on this endotherm also diminishes as *T_cc_*^isothermGLASS^ is raised while its location is shifted at slightly higher temperatures up to 120 °C when it starts to decrease. This higher-temperature endotherm is related to the melting of the recrystallized entities after their thickening on melting. If during isothermal cold crystallization pristine crystals are developed at temperatures high enough, they would become sufficiently thick and the two processes would tend to merge each other and, eventually, only one melting endotherm would be observed, as it happens for PLLA4 (see below). Nevertheless, the highest *T_cc_*^isothermGLASS^ used for PLLA2 has not been enough to lead to this situation of perfection in the α crystals.

The incorporation of SBA-15 silica in PLLA2SBA8 does not seem to affect much the crystalline characteristics derived from the isothermal cold crystallization performed at this temperature interval (see below).

Scenario is quite different if attention is paid into the pristine PLLA4 and its composite PLLA4SBA9. On one hand, the α’ → α transition is not noticeable even at the lowest *T_cc_*^isothermGLASS^ here tested. On the other hand, perfection of neat crystals developed at crystallization temperatures high enough is achieved since the merging into only one melting peak of the two initially evident endothermic events is observed. Even under this most favorable situation, melting temperature does not reach 160 °C because of the lack of chain regularity ascribed to the high *D* isomer content in this PLLA4 matrix. Again, incorporation of mesoporous silica particles does not influence much the location of these two melting peaks, *T_m_*_1_ and *T_m_*_2_ (see below).

**Figure 7:**
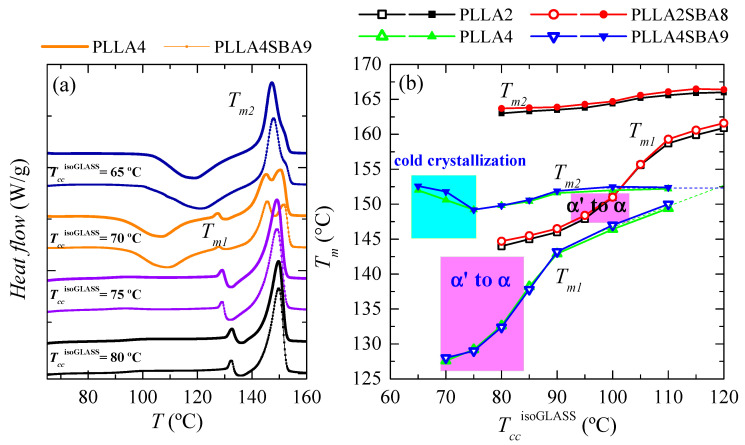
(**a**) Melting behavior as a function of the temperature of isothermal crystallization from the glass, *T_cc_*^isothermGLASS^, for long-time in pristine PLLA4 and its composite PLLA4SBA9. (**b**) Variation of *T_m_*_1_ and *T_m_*_2_ on *T_cc_*^isoGLASS^ for PLLA2, PLLA4, PLLA2SBA8 and PLLA4SBA9.

Comparison of melting curves represented in Figure 6 between both PLLAs and their composites seems to point out a common behavior but shifted in the isothermal cold crystallization temperature scale. Thus, DSC curves of PLLA4 and PLLA4SBA9 within the *T_cc_*^isothermGLASS^ range from 85 to 105 °C are very analogous to those for PLLA2 and PLLA2SBA9 but now in the *T_cc_*^isothermGLASS^ interval between 100 and 120 °C. The α’/α-crystal transformation is not observed at this *T_cc_*^isothermGLASS^ range for the PLLA4 and PLLA4SBA9 specimens and, accordingly, that mentioned similarity only concerns samples when the α polymorph is developed in the majority. However, are PLLA4 and PLLA4SBA9 able to lead to the formation of the α’ crystalline lattice, or is this polymorph prohibited to be developed from isothermal cold crystallization at high *D* isomer content? In order to answer this question, it has to be considered that study of the crystallization isotherm at annealing temperatures below 85 °C is not feasible under these experimental conditions, due to the worsening of the signal-to-noise ratio as a consequence of the rather high required crystallization times (see Figure 5). Thus, additional isothermal cold crystallization experiments were performed for longer times, 8 h, without registering the DSC isotherm. Their subsequent melting curves are shown in Figure 7a.

DSC curves for PLLA4 and PLLA4SBA9 crystallized from the glass at 65 °C exhibit two clear thermal transitions: an extra cold crystallization and a further melting process. The appearance of the dynamic cold crystallization on the heating means that these two materials were not able to finish their ordering from the glassy state by staying for 8 h at 65 °C. Even more, this temperature is so low that crystallization does not take place. Then, DSC enthalpies implied in the cold crystallization and the further melting processes yield a nearly zero neat total enthalpy of melting, i.e., an almost zero degree of crystallinity. Independently of the presence or absence of silica particles, glassy specimens crystallized at 65 °C are basically amorphous. Therefore, the crystals that melt are those developed along the cold crystallization from the glassy state. The location of *T_cc_* at around 120 °C points out that the α phase [34] is the one developed during the DSC heating run.

**Figure 8:**
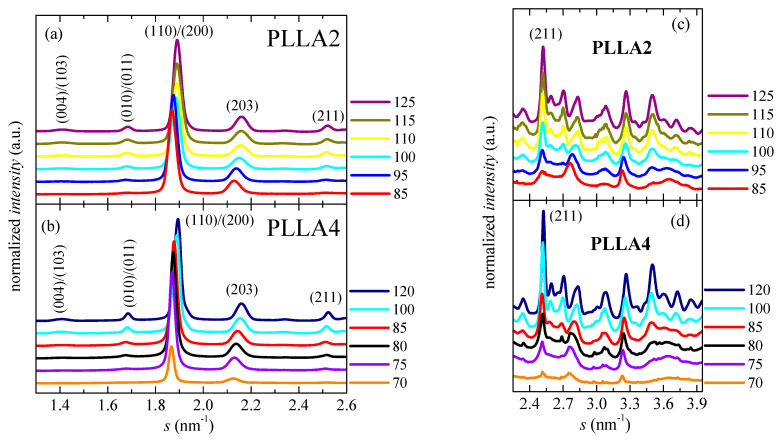
Synchrotron pure crystal WAXS profiles, at room temperature. Scattering intervals: (**a**,**b**) from 1.30 to 2.60 nm^−1^; and (**c**,**d**) from 2.25 to 3.95 nm^−1^, for PLLA2 and PLLA4 after isothermal cold crystallization at different temperatures. The main Miller indices are indicated.

Specimens isothermally cold crystallized at 70 °C show ordering features more complex. Maintenance of these materials at this temperature allows the development of a certain number of crystalline entities, as deduced from their X-ray profiles and from the no zero neat total enthalpy of melting. These initial crystallites are supposed to be α’-type because of the temperature at which they have been formed. This assumption is corroborated by X-ray, whose pure-crystal profile is represented in Figure 8, and by observation of the α’/α-crystal transformation in the DSC curves through the very small endothermic event (at around 128 °C) overlapped with a little exothermic process. Nevertheless, the degree of crystallinity in these initial α’ crystallites is relatively low because a cold crystallization on heating is clearly noted at temperatures lower than the α’ → α transition. This cold crystallization appears at a lower temperature than that observed when samples are crystallized at 65 °C because of the presence of the α’ crystals (developed along isothermal cold crystallization at 70 °C) acting as nucleating agents. These new crystals generated during the DSC heating run are expected to be preferentially α-type. Concerning the main melting process, this is clearly bimodal. The lower-temperature process is associated with the melting of more imperfect crystallites created during cold crystallization, while the more intense high-temperature endotherm is ascribed to those transformed from the α’ polymorph and those able to thicken on melting.

A remarkable change is noted when isothermal cold crystallization is carried out at a slightly higher temperature of 75 °C. A complete ordering has taken place at 75 °C since DSC cold crystallization is not exhibited on heating. The α’ polymorph is developed at this temperature and, accordingly, the α’ to α-crystal transformation is now clearly noticeable in the temperature interval from 130 to 140 °C, being moved to slightly higher temperatures than in the specimens isothermally crystallized at 70 °C. The intensity is also larger. Both features are associated with a higher amount of α’ crystallites. This temperature of 75 °C allows full ordering during isothermal annealing for 8 h and then, a further cold crystallization is not shown in the DSC heating run, as mentioned. Moreover, a unique endothermic peak is seen in the main melting process.

The behavior of the specimens isothermally crystallized at 80 °C is rather similar to that just described for samples treated at 75 °C. A displacement of the transformation from α’ to α crystals to slightly higher temperature is noticed together with a small narrowing of the main melting process.

Figure 7b summarizes the location of *T_m_*_1_ and *T_m_*_2_ for all the samples analyzed as a function of the *T_cc_*^isothermGLASS^ employed. The influence that the *D* isomer content exerts on these melting temperatures is evident while the presence of SBA-15 turns out insignificant in the melting event in spite of its key role in the crystallization processes, either from the molten or glass state. In addition to the lower values (around 12–15 °C lower) for *T_m_*_1_ and *T_m_*_2_ observed in PLLA4 and PLLA4SBA9 due to the smaller chain regularity, other features can be deduced. On one hand, the temperatures at which the α’ polymorph is developed during isothermal crystallization from the glass are also considerably lower in PLLA4 and PLLA4SBA9 than in PLLA2 and PLLA2SBA8, being in the former from 70 to 80 °C (since the majority of crystals at 85 °C are α-type [58]) while its development in PLLA2 and PLLA2SBA8 takes place in the range from around 95 to 105 °C. This lowering of temperatures makes the formation interval of α’ crystals in the PLLA4 overlap with the glass transition.

On the other hand, the parameter *T_m_*_2_, appearing at the highest temperature, is sensitive to the just mentioned behavior through the existence of an additional cold crystallization during the DSC experiment. PLLA chain mobility is insufficient to allow global ordering when isothermal crystallization from the glass state occurs close to the glass transition temperature. Then, the formation of a three-dimensional arrangement does not take place (as happened in PLLA4 and PLLA4SBA9 at 65 °C) or appears to a low extent and the ordering has to be completed during the heating DSC run through a cold crystallization. The α crystals developed along that cold crystallization are rather imperfect and thin, leading to a decrease in the *T_m_*_2_ value (see the cyan rectangle in Figure 7b).

Anyway, DSC melting curves represented in Figure 7a have confirmed that PLLA4 and its composite PLLA4SBA9 are able to develop the α’ polymorph. Its crystallization, however, takes place very close to the PLLA glass transition, thus requiring very long crystallization times.

Crystalline details for PLLA2 and PLLA4 obtained from isothermal cold crystallization are analyzed by X-ray diffraction in order to examine the influence of the *D* content. The evaluation is only performed on neat PLLAs since the influence of SBA-15 particles has turned out only slight during the melting processes after isothermal cold crystallization, as deduced from Figure 6 and Figure 7b. Figure 8 shows the pure crystalline profiles (after subtracting the corresponding amount of the amorphous component) for the two pristine PLLAs after their crystallization from the glass at different temperatures. The amorphous halo used for obtaining these pure crystal components was the diffractogram corresponding to the original compression-molded film of PLLAs (analogous to those represented in Figure 3b), which is totally amorphous.

**Figure 9:**
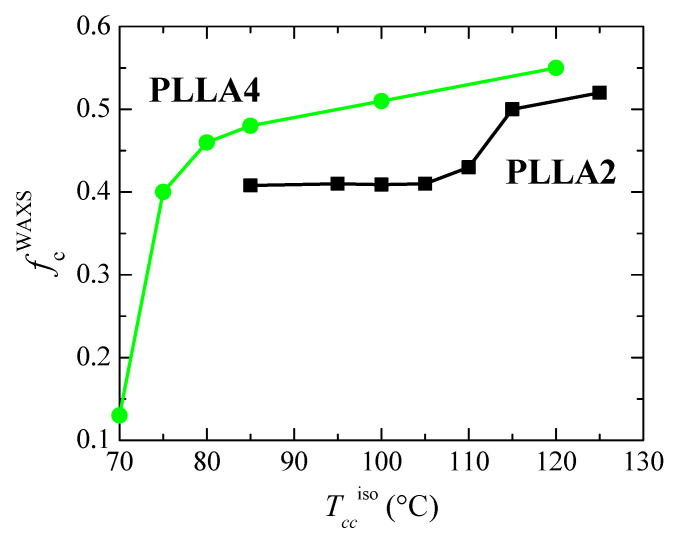
Dependence on crystallization temperature, *T_cc_*^iso^, of the degree of crystallinity determined by WAXS for the neat PLLAs studied.

The degree of crystallinity can be, therefore, easily estimated from these pure crystalline WAXS profiles, as depicted in Figure 9. These WAXS patterns in Figure 8 have been represented at two different scattering ranges for a better visualization: that involving the most intense diffractions (110)/(200) and (203) (on the left) together with another displaying the multiple weak reflections appearing at higher scattering values (on the right). This latest section of the diffractograms (Figure 8c,d) has been considerably amplified in the Y scale, as deduced from the intensity of the (211) reflection shown in both representations.

As previously commented, the α’ crystals are reported to be developed at temperatures lower than about 120 °C [31]. Figure 8 indicates that temperature ranges where α′ and α lattices grow are, however, strongly affected by *D* content existing in the PLLAs evaluated. Cold crystallization for just some minutes at 85 and 95 °C in PLLA2 leads to the α’ form, as deduced from the location of its main (100)/(200) and (203) reflections. As expected, they appear at scattering values lower than those for the α modification. Furthermore, the characteristic diffraction of α’ phase at 2.76 nm^−1^ is noticeable [46]. Cold crystallization at temperatures higher than 95 °C goes faster in this PLLA2 (see Figure 5), being the α cell the major modification formed. Therefore, the position of the (100)/(200) and (203) diffractions remains rather constant and only an enlargement in their intensity takes place due to the rise of crystallinity with increasing cold crystallization temperatures, which is more evident above 105 °C, as deduced from Figure 9. The highest value of crystallinity reached in PLLA2 under these crystallization conditions is 0.52.

Crystalline characteristics found in PLLA4 are quite dissimilar to those described for PLLA2. First of all, it has to be considered that temperature and time intervals are different due to the hindrance for cold crystallization in PLLA4 ascribed to its highest content in *D* isomer, which considerably reduces its chain regularity and, accordingly, its global crystallization capability. This implies particularly the formation of the α’ polymorph. It only crystallizes, as seen above, at very low temperatures, close to its *T_g_*, requiring very long isothermal cold crystallization times. In fact, a small amount of α’ phase is achieved after annealing for 15 h at 70 °C, as noted in Figure 8b for the pure crystal WAXS profile at that temperature. Crystallinity achieves the value of only 0.13, as depicted in Figure 9. The α’ polymorph is also the main lattice developed at 75 and 80 °C, as revealed by observation of its distinctive reflection at 2.76 nm^−1^ [46] in Figure 8d, although at 80 °C it could be coexistence with a minor amount of α crystallites. Figure 8b shows, at these low temperatures, a slight displacement to higher *s* values and an increase in intensity for the main (100)/(200) and (203) reflections, both features associated with the improvement and rise in crystallinity of those α’ crystallites. A further increase in temperature moves the location of these primary diffractions to even higher *s* values, but now the main modification generated is the α lattice, as deduced from the disappearance of reflection at 2.76 nm^−1^ only ascribed to the α’ form [46] and from observation of (004)/(103), (204) and (207)/(117) diffractions exclusively related to the α polymorph [59]. Continuous evolution of crystalline characteristics is noticed in PLLA4 along the cold crystallization temperature range: perfection of α’ lattice and increase in its amount up to 80 °C; α’ → α transition from 70 to 85 °C (the small exotherm in the heating DSC curve is no longer exhibited at 85 °C, as seen in Figure 6c); and, thickening of α crystals as well as an enlargement in crystallinity above 85 °C. These progressive changes are associated with the crystallization difficulty found in PLLA4 because its around double molar fraction in *D* isomer compared with that existing in PLLA2. Despite this hindrance, a remarkable crystallinity is reached. These values even become in PLLA4 larger than in PLLA2 under the crystallization conditions imposed, although it should be reminded that the protocol applied to PLLA4 has involved much longer crystallization times and, eventually, considerably much more favorable conditions.

**Figure 10:**
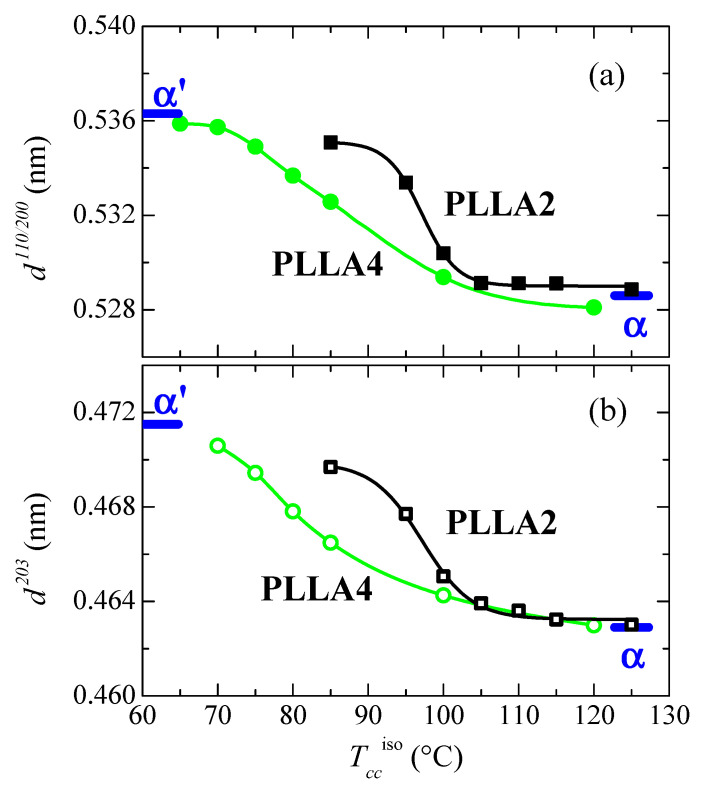
Variation with *T_cc_*^iso^ of the PLLA2 and PLLA4 spacings, at room temperature, for (**a**) the (110/200) diffraction; (**b**) the (203) diffraction (see Figure 8a,b). The thick marks are the corresponding values described in ref. [32] for the α’ and α modifications.

Figure 10 represents the dependence of location for the main reflections on crystallization temperatures. Variation in the position is related to the α’ → α transition due to the similarity that both lattices show in these main diffractions. Taking into consideration the values previously reported for these two neat polymorphs [32], displacement of their positions goes from 0.5363 to 0.5286 nm^−1^ for the (110)/(200) reflection and from 0.4715 to 0.4629 nm^−1^ for the (203), when comparing the α’ form with the α modification. The higher order exhibited by the α phase is responsible for the location of these two primary diffractions at inferior spacings compared with those for the α’ form due to the higher compactness of the former. Figure 10 also displays that the α’ → α transition seems to occur for PLLA2 in a temperature range narrower than for PLLA4. The higher lack of chain regularity in PLLA4 due to its much higher content in *D* isomer slows down the rate in the transition between these two crystalline modifications and, accordingly, temperature interval becomes broader. Its location is also dependent on the *D* molar fraction, estimated to take place at approximately 97.5 °C in PLLA2 while it is seen at around 82.5 °C in PLLA4.

**Figure 11:**
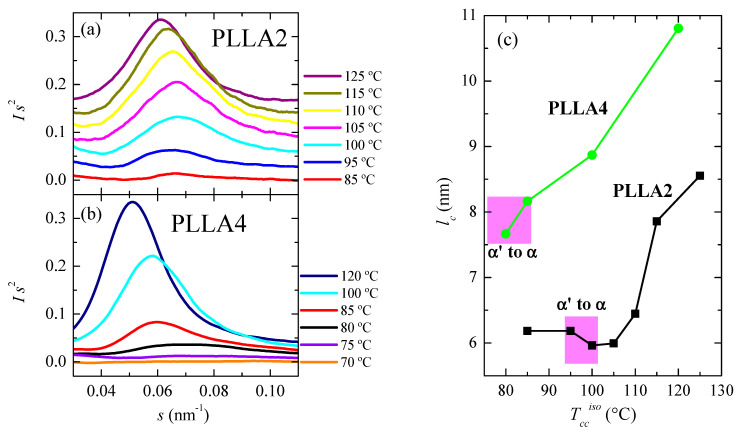
SAXS profiles, at room temperature, for different cold crystallized temperatures: (**a**) for PLLA2 and (**b**) for PLLA4. (**c**) Dependence of most probable crystallite size on isothermal cold crystallization temperature for PLLA2 and PLLA4, estimated from SAXS long spacing values assuming a two-phase model (*l_c_* = *L*^SAXS^ × *f_c_*^WAXS^).

Another important magnitude analyzed has been the crystal thickness. Figure 11a,b shows the synchrotron SAXS 1D profiles at room temperature for some of the samples. Long spacing, *L*, is only noticeable in these curves when isothermal cold crystallization has taken place at 80 °C or temperatures above. That means that electron density differences between the two existing phases, amorphous and crystalline, are usually not high enough in the α’ form of PLLA4 to lead to the observation of long spacing. Only when quite perfect α’ crystals are developed and in a sufficient amount, its chain conformation and packing mode allow reducing its intrinsic lateral disorder and improving the electron density contrast, making possible the appearance of a small-intensity long spacing. This is a valuable parameter in the lamellar stack model theory for semicrystalline polymers since it exhibits considerable changes during crystallization, recrystallization, or melting processes in those semicrystalline polymers [60,61,62,63].

Values of scattering vector, *s*, for the maxima in the SAXS profiles of PLLA2 are higher than those displayed by PLLA4, for the analyzed crystallization temperatures. Consequently, the *L* parameters are lower since *L* = 1/*s*. This feature is again associated with the much longer crystallization times employed for PLLA4, leading, as shown above in Figure 9, to higher crystallinities in the case of PLLA4, in spite of its higher content in *D* isomer. Consequently, most probable crystallite sizes, estimated from SAXS long spacing values assuming a two-phase model (*l_c_* = *L*^SAXS^ × *f_c_*^WAXS^), and represented in Figure 11c, become larger in PLLA4 than those achieved in PLLA2. Moreover, it follows from this Figure that the crystal thickness is clearly sensitive to the presence of the two different crystalline forms, and to the transition between them.

Microhardness measurements performed at room temperature provides information about the mechanical behavior exhibited by these two PLLAs just after their processing. Effect of content in *D* isomer together with that caused by incorporation of SBA-15 particles is then analyzed on the pure amorphous PLLA macrochains (see Figure 3b) for different times. This initial amorphous state has been selected due to the important differences just described in the crystallization capabilities of both PLLAs analyzed, ascribed to the variation in the content of *D* isomer.

**Figure 12:**
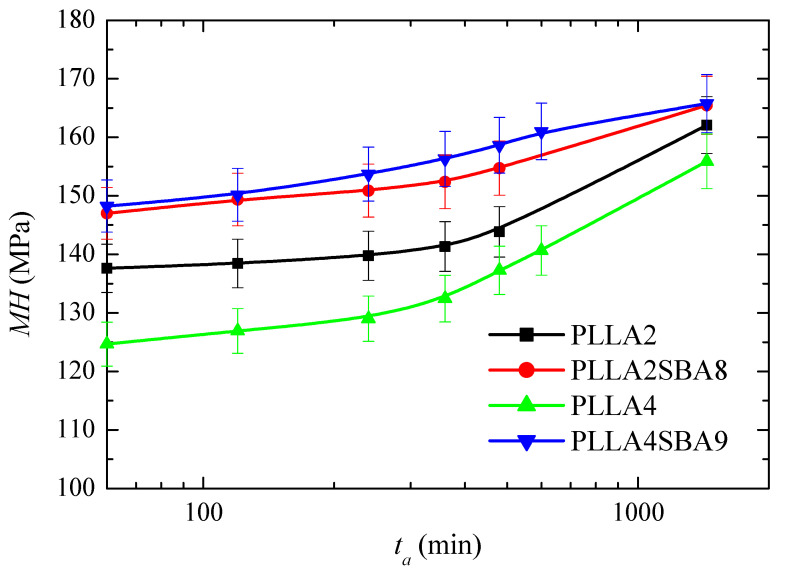
Microhardness, *MH*, dependence on aging time (*t_a_*) for the neat amorphous PLLAs (PLLA2 and PLLA4) and for their amorphous composites with mesoporous SBA-15 particles (PLLA2SBA8 and PLLA4SBA9) just after processing from the melt.

PLLA glass transition is around 60 °C although is slightly higher in PLLA2 and PLLAS2BA8 than in PLLA4 and PLLA4SBA9, as deduced from Figure 3e. Consequently, PLLA can undergo at room temperature physical aging in its amorphous regions. This phenomenon is related to molecular rearrangements that take place within the structure of PLLA chains. Accordingly, its thermodynamic variables (enthalpy, entropy, and volume) evolve toward equilibrium [64,65] during the physical aging process. This progress until equilibrium is reached provokes changes in the entire spectrum of properties with time (aging time). Four weeks is considered for the amorphous PLLA enough time [17] to attain equilibrium at room temperature. This process could be sped up if temperatures closer to *T_g_*, but still below, are used [64]. Figure 12 shows the microhardness (*MH*) data for both PLLAs analyzed and their composites, PLLA2SBA8 and PLLA4SBA9, with mesoporous silica at several aging times.

*MH* value in PLLA2 after 60 min from its processing is higher to that obtained from PLLA4, which is attributed to the higher compactness of its amorphous macrochains because its lower amount in *D* isomer compared with those existing in PLLA4. This trend is exhibited in the entire range of aging time analyzed: up to 24 h after processing. The *MH* variation is similar for both PLLAs in this interval and, thus, an increase of values is observed with rising aging times, as expected, because of densification of their amorphous chains [66]. Volume is diminished with aging time, then PLLA chains become more compact and, consequently, more rigid during this process. All the mechanical parameters associated with stiffness increase, therefore, with aging time.

The incorporation of mesoporous silica provokes an increase in *MH*, independently of using PLLA2 or PLLA4. Accordingly, the reinforcement effect of SBA-15 particles, which are much more rigid, is clearly noticeable in both of them, with considerably higher values of *MH* in relation to the PLLA alone. The increase is, however, much higher in the case of PLLA4SBA9. Thus, the initial increase is around 23 MPa for this PLLA4SBA9 while it is only around 10 MPa for PLLA2SBA8. As a consequence, the two composites show analogous values at the different aging times, within the experimental error, although PLLA4SBA9 seems to present even slightly higher *MH* data. This feature indicates that the addition of a similar content of SBA-15 particles leads to a more important filler effect in the PLLA4 matrix than in the PLLA2. This higher improvement in the *MH* of PLLA4 by the presence of SBA-15 silica is able to compensate for its initial softer nature because of its high content in *D* isomer, turning out more significant the inclusion of a stiffer component.

## 4. Conclusions

Two *L*-rich PLLAs with distinct contents in *D* isomer and their composites with an intermediate amount of mesoporous SBA-15 (at about 9 wt.%) were processed by melt extrusion. Important microstructural differences are ascribed to the distinct *D* isomer content, around 2.0 and 4.2, respectively, since molecular weights and polidispersities are rather analogous. Appropriate distribution of silica particles was found in both PLLA matrices, independently of its *D* content, together with the no appearance of bulky inorganic domains.

The incorporation of SBA-15 particles in these two PLLA matrices did not play a clear effect in the thermal degradation of these PLLA2SBA8 and PLLA4SBA9 composites.

PLLA cold crystallization on dynamic heating runs was strongly dependent on the *D* content in the neat matrices. This process was much broader in PLLA4 than in PLLA2, indicating the much more hindrance found for ordering in the former. The difference becomes even larger when SBA-15 particles are incorporated, these playing a role of nucleating agents.

Isothermal crystallization from the melt showed that incorporation of SBA-15 silica sped up the PLLA crystallite formation in the composites, independently of the content in *D* isomer. Mesoporous silica was found to nucleate more favorably in PLLA4 than in PLLA2.

Isothermal PLLA crystallization from the glass and the subsequent melting processes also exhibited differences in the cold crystallization temperature window between PLLA2 and PLLA4 and their composites because of their dissimilar content in *D* isomer. The formation of α’ polymorph was hampered for the PLLA4 and PLLA4SBA9 specimens, being developed at temperatures slightly higher than their glass transition, from 70 to 80 °C, while its development in PLLA2 and PLLA2SBA8 samples was possible in the range from 80 to 105 °C. Accordingly, observation of the α’/α-crystal transformation occurs in PLLA4 and PLLA4SBA9 at temperatures much lower than in the PLLA2 and PLLA2SBA8 materials.

Appearance of a small, long spacing was evident only when quite perfect α’ crystals were developed and in a sufficient amount. For the α crystals, however, well-defined intense long spacings were observed in all cases. Very significant values of crystallinity and most probable crystal size can be reached if appropriate conditions (time and temperature) were selected.

Finally, the reinforcement effect of SBA-15 silica in the amorphous materials (just after they were processed) was more considerable in the softest initial sample: PLLA4.

To summarize, PLLA properties are deeply dominated by its content in *D* isomer. Before the definition of a particular application, a thorough evaluation is required for a specific PLLA grade to learn its crystallization characteristics (temperature interval, crystallinity values, the ratio between α’ and α polymorphs, crystallite size) since the whole properties spectrum is controlled by them. Moreover, SBA-15 particles exhibit important nucleation ability in the PLLA grades analyzed. Exterior and interior surfaces of these mesoporous silica can be readily modified, fact that could constitute an interesting tool for incorporation of tailored functionalities into PLLA based materials allowing extending their applicability in environmental, biomedical, or energy fields, among others.

## Figures and Tables

**Table 1:** Names of samples analyzed; molar % in *D*-lactic acid units; molecular mass and polydispersity results of the used PLLA grades estimated after their extrusion.

Sample	Commercial Trademark	*D*-Isomer Content (mol %)	*M_w_* (g/mol)	*M_w_/M_n_*
PLLA2	Ingeo6202D	2.0 ± 0.20	118,600	1.6
PLLA4	PLA3051D	4.2 ± 0.40	117,600	1.8

**Table 2:** TGA results under inert and oxidative environments for neat PLLAs and their composites attained by melt extrusion: temperature of the 10% loss mass (*T*_10%_) and maximum temperature (*T*_max_) together with the SBA-15 wt.% amount estimated from both atmospheres and its average.

Sample	Average SBA-15 wt.% Content	Inert Atmosphere			Oxidative Atmosphere		
		*T*_10%_ (°C)	*T*_max_ (°C)	SBA-15 wt.% Content	*T*_10%_ (°C)	*T*_max_ (°C)	SBA-15 wt.% Content
PLLA2	0	328.0	363.5	0	332.5	364.5	0
PLLA2SBA8	8.2	328.5	355.0	8.5	334.5	359.5	7.8
PLLA4	0	328.0	361.0	0	333.0	357.0	0
PLLA4SBA9	9.3	343.0	369.5	9.6	336.5	362.5	8.9

## Data Availability

Data are contained within the article.
